# Habitat geometry rather than visual acuity limits the visibility of a ground‐nesting bird's clutch to terrestrial predators

**DOI:** 10.1002/ece3.10471

**Published:** 2023-09-14

**Authors:** George R. A. Hancock, Lizzie Grayshon, Ryan Burrell, Innes Cuthill, Andrew Hoodless, Jolyon Troscianko

**Affiliations:** ^1^ Centre for Ecology and Conservation University of Exeter Penryn UK; ^2^ Game and Wildlife Conservation Trust Fordingbridge UK; ^3^ Faculty of Science and Technology Bournemouth University Dorset UK; ^4^ School of Biological Sciences University of Bristol Bristol UK

**Keywords:** 3D scanning, camouflage, ground‐nesting birds, lapwing, occlusion

## Abstract

The nests of ground‐nesting birds rely heavily on camouflage for their survival, and predation risk, often linked to ecological changes from human activity, is a major source of mortality. Numerous ground‐nesting bird populations are in decline, so understanding the effects of camouflage on their nesting behavior is relevant to their conservation concerns. Habitat three‐dimensional (3D) geometry, together with predator visual abilities, viewing distance, and viewing angle, determine whether a nest is either visible, occluded, or too far away to detect. While this link is intuitive, few studies have investigated how fine‐scale geometry is likely to help defend nests from different predator guilds. We quantified nest visibility based on 3D occlusion, camouflage, and predator visual modeling in northern lapwings, *Vanellus vanellus*, on different land management regimes. Lapwings selected local backgrounds that had a higher 3D complexity at a spatial scale greater than their entire clutches compared to local control sites. Importantly, our findings show that habitat geometry—rather than predator visual acuity—restricts nest visibility for terrestrial predators and that their field habitats, perceived by humans as open, are functionally closed with respect to a terrestrial predator searching for nests on the ground. Taken together with lapwings' careful nest site selection, our findings highlight the importance of considering habitat geometry for understanding the evolutionary ecology and management of conservation sites for ground‐nesting birds.

## Graphical abstract

We used 3D scanning and color‐calibrated images to investigate the effects of local geometry on ground‐nesting bird site selection and camouflage. We found that lapwings, *Vanellus vanellus*, locally select for more 3D variable areas and that the scale and shape of their nests restrict visibility more than predator visual acuity, with crop fields and wet grassland acting more like a closed habitat from the viewing angles of natural predators.

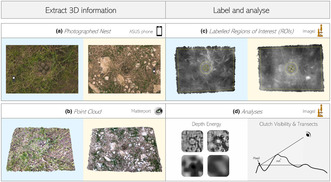

## 1 INTRODUCTION

Camouflage is one of the most common anti‐predator strategies exhibited by animals, as reducing the ability of predators to detect or distinguish a target from its background reduces the risk of predation (Cott, 1940; Cuthill, 2019; Endler, 1981). Ground‐nesting birds are no exception to this, with many of their species exhibiting camouflage at various phases in their life history (Stevens et al., 2017; Stoddard et al., 2016). One notable phase where camouflage has evolved is that of the egg (Kilner, 2006; Westmoreland, 2008). The comparative openness and accessibility of ground‐nesting wader nests, such as those of coursers (Cursoriinae) and plovers (Charadriinae), renders them particularly vulnerable to predation. When predators approach, adult coursers and plovers abandon their nests (Blumstein, 2003; Wilson‐Aggarwal et al., 2016), relying on the patterns of their eggs to camouflage them while the parent(s) harass or distract the predator (Armstrong, 1954; Simmons, 1951). The camouflage of these ground‐nesting birds eggs varies from species to species depending on their nesting behavior. Nightjars, which rely more on the parents' plumage for camouflage, have been shown to have poorer egg camouflage compared to more open‐nesting plover and courser species (Wilson‐Aggarwal et al., 2016). In other ground‐nesting species, the eggs can be occluded by either burying them, or by relying on vegetation from surrounding hedgerows, scrub, or forest understory (Amat et al., 2012; Bravo et al., 2022; Stevens et al., 2017; Stoddard et al., 2011; Troscianko, Wilson‐Aggarwal, Spottiswoode & Stevens, 2016).

Hiding behind natural structures is arguably one of the most effective ways for prey to evade detection. Total occlusion forces observers to rely on other sensory cues, that is, olfaction and audition, to detect the occluded object, provided that the source of occlusion is not also recognizable, for example, nesting material or the incubating parent (Bailey et al., 2015; Broughton & Parry, 2019; Stevens et al., 2017). Meanwhile, partial occlusion can aid camouflage by masking important visual cues for detection and recognition such as an object's outline, size, and identifiable morphological features (limbs, eyes, etc.) (Bailey et al., 2015; Broughton & Parry, 2019; DiPietro et al., 2002; Sharman et al., 2018; Stevens et al., 2017; Sovrano & Bisazza, 2008; Tvardíková & Fuchs, 2010). Nevertheless, the degree of partial occlusion required to interfere with these recognition mechanisms and any interactions with background appearance remain unknown. While predators can use olfaction and auditory cues to detect occluded prey, existing experiments with trained dogs and foxes have shown them to struggle to detect nests with nonvisual cues (Seymour et al., 2003; Storaas et al., 1999). There is also evidence of active camouflage of sound and smell by silencing prenatal calls in response to predators and by changing to odorant production during incubation (Grieves et al., 2022; Kostoglou et al., 2021). These experiments highlight the importance of vision in ground‐nesting bird nest detection.

The nesting‐ecology of ground‐nesting birds often involves trade‐offs between biotic and abiotic factors that affect the survival of parents and/or their offspring, such as predation (Troscianko, Wilson‐Aggarwal, Stevens & Spottiswoode, 2016), thermoregulation (Amat et al., 2012; Kubelka et al., 2019), and other habitat‐linked risks (e.g. trampling, flooding; Wilson et al., 2001). Nest occlusion is a key strategy with a diverse range of solutions. Burrow‐nesting birds achieve full occlusion, while others use tall, thick vegetation to achieve occlusion, such as snipe and redshank. However, occlusion also has costs, as it prevents parents from seeing approaching predators. Indeed, cover by surrounding vegetation has been found to influence parent predation in addition to nest thermoregulation and both habitat and nesting material availability (Amat & Masero, 2004; Gillis et al., 2012; Kubelka et al., 2019; Mainwaring et al., 2014; Stevens et al., 2017; Swaisgood et al., 2018). As such, many species nest on bare, flat ground. Vegetation height has been shown to influence nest site selection, mortality (Gómez‐Serrano & López‐López, 2014), and body condition (Amat & Masero, 2004). Taller vegetation results in greater nest survival and reduced adult temperature exposure in warmer climates, but shorter flushing distances and greater parent predation risk due to the occlusion of predators (Amat & Masero, 2004; Bertholdt et al., 2017; Gómez‐Serrano & López‐López, 2014; Wilson‐Aggarwal et al., 2016). While most studies assess vegetation's effect on visibility using height alone, Gómez‐Serrano and López‐López (2014) used periscopes to assess the visibility of predators (dogs and humans) from the perspective of nesting Kentish plovers (*Charadrius alexandrines)*. They found sites selected by parents offered greater predator visibility at the cost of increased nest predation risk (Gómez‐Serrano & López‐López, 2014). This study, however, did not look at how occlusion influenced clutch visibility from the predator's perspective.

When measuring nest camouflage, the visual ecology of the observing parents and predators should be considered. Visual modeling using color‐calibrated images has increasingly been used to assess animal camouflage from different visual systems, accounting for differences in observer color reception and spatial acuity (Caves et al., 2018; Maia et al., 2013; van den Berg et al., 2020). These measures have been used to show that camouflage from local background patterns can predict nest survival in ground‐nesting birds (Troscianko, Wilson‐Aggarwal, Stevens & Spottiswoode, 2016). However, an aspect of visual ecology rarely considered is predator height in combination with distance and habitat structure. The height of an animal's eye relative to its objects of interest changes the angles and distances required for said objects to be unoccluded by surrounding structures (Martin, 2011). A nest that appears exposed from a human height may be entirely obstructed when viewed by a smaller mammalian predator, even at closer distances, while an avian predator excluded to the edge of a field by harassing parents may be at too great a distance to resolve a clutch of eggs (Gómez‐Serrano & López‐López, 2014). Microhabitat selection likely helps balance the trade‐offs between predator and nest visibility (Gómez‐Serrano & López‐López, 2014; Lovell et al., 2013; Stoddard et al., 2016). Most open ground‐nesting bird nests comprise a shallow, lined depression in the ground, referred to as a scrape. Scrapes have been shown to help insulate clutches and are likely to also aid in keeping the individual eggs together (Tulp et al., 2012). By selecting areas that are even slightly elevated compared to the local surroundings, ground‐nesting birds should be able to increase their field of view for detecting predators. Combined with the depression of the scrape, local elevation should paradoxically decrease nest visibility from terrestrial predators, requiring a greater viewing angle for an unobstructed view.

Ground‐nesting waders are in decline across their range due to habitat loss, agricultural intensification, reduced prey availability, and elevated predation risk from mesopredators, such as foxes, mustelids, corvids, and raptors (Evans, 2004; Galbraith, 1988; Roos et al., 2018; Vickery et al., 2004). Consequently, mechanisms for further understanding the habitat features that both encourage nesting and minimize predation are of increasing conservation interest, as predation is typically the leading cause of nest mortality (Baines, 1990; Ricklefs, 1969; Teunissen et al., 2008). Just as camera quality has advanced color analyses of visual scenes, the increasing accessibility of terrestrial and aerial 3D scanners allows for the measurement of topography and vegetation structure at different spatial scales (de Vries et al., 2021; Hill et al., 2014; Li et al., 2022). Terrestrial scanners have even been used to compare the volume and shape of bowl‐nesting birds, though these were taken in vitro (Simonov & Matantseva, 2020). 3D scanning allows for a more complete measure of local 3D composition than more traditional Munsell Soil Charts or ruler‐based measurements of vegetation height and cover (Gómez‐Serrano & López‐López, 2014; Gregg, 1991; Pendleton & Nickerson, 1951).

In this study, we used hand‐held 3D scanners and color‐calibrated images to measure the shape and appearance of northern lapwing (*Vanellus vanellus*) nests in pastoral, arable, and wet grassland sites. The goal was to investigate how the 3D and color environments influence lapwing nesting decisions. The methods of habitat management and local variation in microhabitat structure should also influence the color and 3D composition of the nests. Any changes to vegetation and topography will affect clutch occlusion and the color and geometric match of the clutches to their background. We hypothesized that lapwings should favor backgrounds of higher local elevation, and greater surrounding 3D variation at scales similar to the size of their nests, which are more obstructed from the perspectives of their predators. We also compared the distances where modeled occlusion and acuity influence detectability by predators and investigated whether camouflage from background match and/or occlusion could predict predation in lapwings.

## 2 MATERIALS AND METHODS

A full breakdown of the 3D scanning methods and scripts required is provided within our Data S1, including methods for using photogrammetry‐generated point clouds in place of 3D scanners.

### 2.1 Study system

The northern lapwing (*Vanellus vanellus*) is a ground‐nesting wader that commonly breeds in lowland wet grassland and arable sites across temperate Eurasia (Cramp & Brooks, 1992). The species is of conservation concern in the United Kingdom and mainland Europe, as their populations have been in decline since the 1970s (Wilson et al., 2001). Unsustainable nest predation is cited as a barrier to population recovery (Evans, 2004; Laidlaw et al., 2021; Roos et al., 2018). Northern lapwings nest in more open habitats, typical of plover species. A lapwing nest consists of a shallow scrape in bare ground or short mixed vegetation, lined with varying amounts of dead plant material for insulation (Kubelka et al., 2019). Lapwing nests are defended from predators by using a combination of mobbing, distractive displays, behavioral crypsis, and egg camouflage (Salek & Cepáková, 2006), while positioning away from trees and around waterbodies also protects nests (Eglington et al., 2009; Kaasiku et al., 2022).

We sampled lapwing nests from sites in two separate locations actively monitored by the Game and Wildlife Conservation Trust (GWCT); the Avon Valley in Hampshire [50.93105, −1.78462] and Burpham in Sussex [50.87198, −0.51812]. For individual site coordinates, please contact the GWCT. The Avon Valley sites included a variety of habitats, predominantly under UK agri‐environment schemes, such as wet grassland, marshland, pasture, and a restored ex‐gravel quarry. Conversely, the Sussex sites consisted of arable fields in various stages of rotation between plough, spring cereal, and fallow. Nests were located through communication with local landowners and field surveys. The time of nest outcomes (hatched, abandoned, flooded, trampled, and predated) was monitored using iButton (Thermochron iButton, Maxim Integrated Products, Inc.) nest temperature loggers and weekly nest checks until the point of failure or hatching, following the methods of Hartman and Oring (2006) and Laidlaw et al. (2015). Relative stability of nest temperature during incubation indicated whether a nest was active compared to the drop and subsequent fluctuation in nest temperature caused by hatching, abandonment, or failure. Predated eggshell fragments or the disappearance of clutches or eggs prior to egg weight estimates and hatch dates were encoded as predation events. All sites had some form of predator control or management to protect wading birds. These varied in intensity and included deterrents such as electric fences and crow scarers and removal methods such as Larsen traps, tunnel traps, and shooting (Fletcher et al., 2010; Laidlaw et al., 2021; Malpas et al., 2013).

### 2.2 Ethics statement

Corresponding permissions were granted as part of a collaboration with the GWCT and were approved by the University of Exeter Ethics Committee.

### 2.3 3D scanning and calibrated photography

**FIGURE 1:**
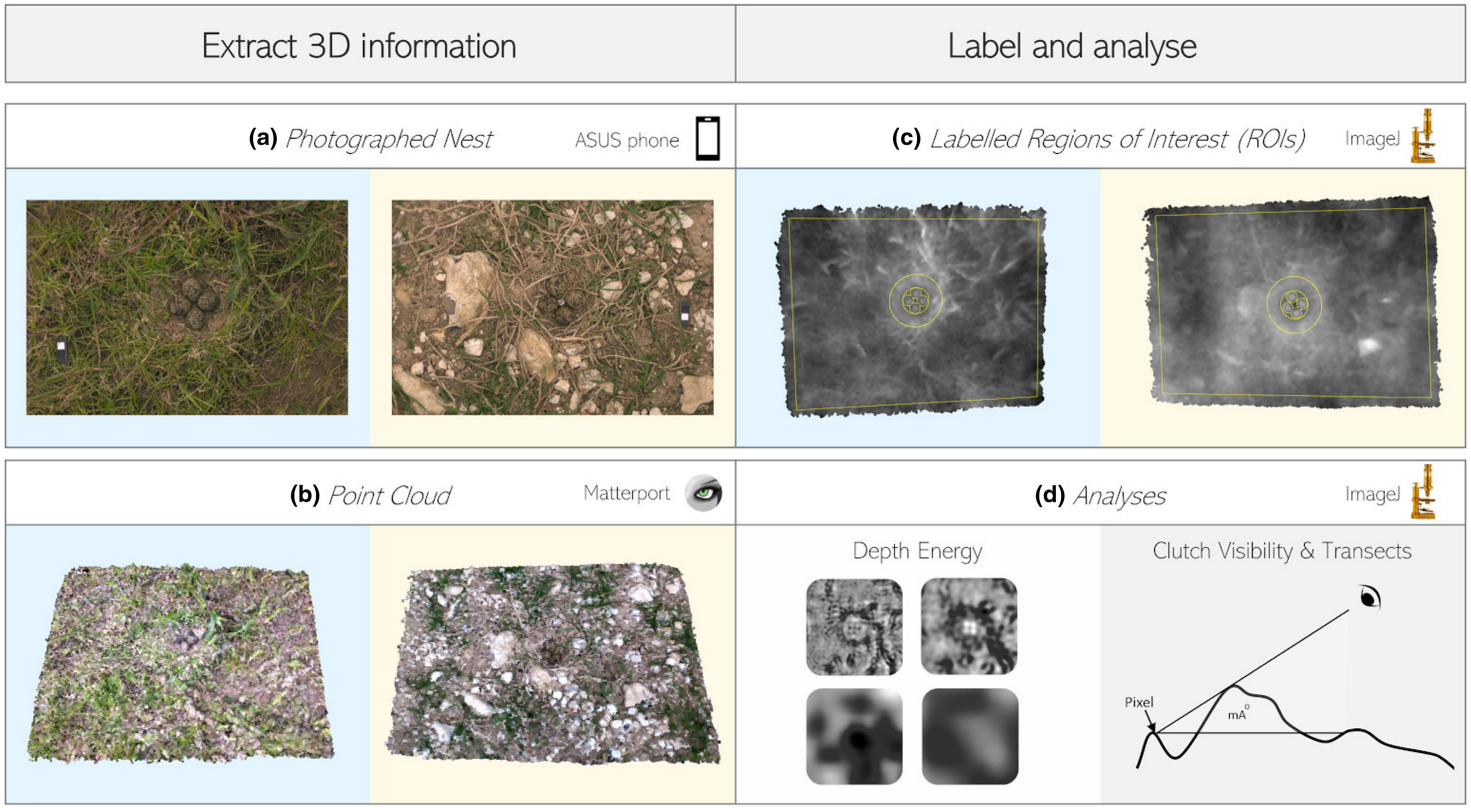
Framework for 3D analyses of ground‐nesting bird nests. Example images are from an agri‐environment scheme site (left‐blue) and spring‐cereal arable site (right‐yellow). (a) Photograph nests with color‐calibrated Sony camera. (b) Create 3D scans of nests with ASUS phone and upload the point cloud into Matterport scenes for standardization and reformatting. (c) Import and label point clouds in ImageJ. (d) Run nest 3D energy and visibility transect analysis scripts.

From March to mid‐June of 2021 and 2022, we photographed 115 lapwing nests and 3D scanned 83. The nests were scanned with an ASUS Zenfone AR using the Matterport Scenes app from a height of 1.2 m (Shults et al., 2019). Phone 3D scanners provide a cheap and relatively easy method for capturing 3D point clouds using triangulation from a structured light time‐of‐flight sensor (Froehlich et al., 2017). Scans and photographs were taken from a height of 1.2 m at a flat 90° (vertical) angle from the ground (Figure 1). To complete one 3D scan, only 7 s are required. For each nest, an additional nest‐less photo and scan were taken at a distance of 1–2 m (4 paces) from the nest, by backtracking in the direction of the approach to avoid further trampling the surrounding area. These additional photos and scans were used as paired nulls for each nest.

Nests were photographed following a protocol based on Troscianko, Wilson‐Aggarwal, Spottiswoode and Stevens (2016) and Troscianko, Wilson‐Aggarwal, Stevens and Spottiswoode (2016). Photographs of the nests and nulls were taken using a chart color‐calibrated Sony A6000 with a Baader venus‐u 52 mm UV filter and the camera's own visible light filter (Moher Alsady et al., 2016). A 7% and 93% uniform (λ 200–700 nm) reflectance standard was placed in situ for each photograph (Troscianko, Wilson‐Aggarwal, Stevens, & Spottiswoode, 2016). Standards were created using Zenith Polymer‐sintered PTFE sheets. Color‐calibrated photography allows for relatively cheap and fast acquisition of spatio‐chromatic information within the environment, while visual modeling allows for the measurement of achromatic and opponent colors for different observer visual systems (Stevens et al., 2007; van den Berg et al., 2020). As the lighting environment was highly variable due to changes in solar angle and weather, all photos were taken with a 1 m^2^ pop‐out NEEWER diffuser sheet at times greater than 2 h from dawn and dusk to prevent patterns from shadows changing the luminance and color measurements of the clutches and their backgrounds (Duarte et al., 2018; Szala et al., 2023; Troscianko, Wilson‐Aggarwal, Stevens, & Spottiswoode, 2016). Photographs were converted to standardized multispectral images using the “generate multispectral image” function within the MICA toolbox v2.2.2 for ImageJ (Schneider et al., 2012; Troscianko & Stevens, 2015; van den Berg et al., 2020).

### 2.4 Constructing height maps

**FIGURE 2:**
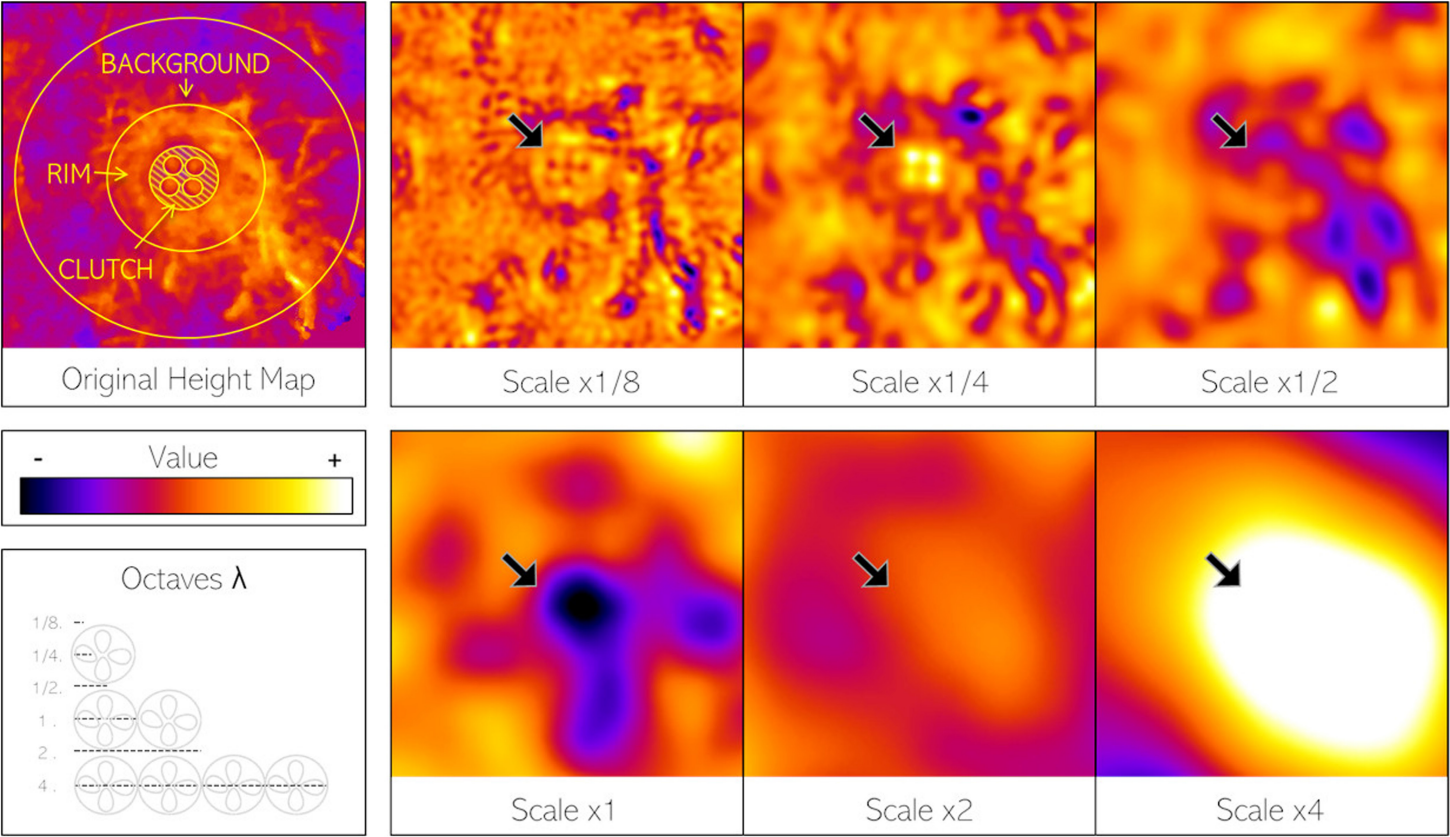
3D scan energy at different spatial scales relative to clutch size: x1/8, x1/4, x1/2, x1, x2, x4. The left‐hand side shows the original height map and associated regions of interest (ROIs): clutch, nest, and background. More elevated (positive) regions are shown as lighter, while less elevated regions are shown as darker. The right‐hand images show the separated spatial scales with the position of the clutch marked by a black arrow. The individual eggs are most visible at x1/4 scale, the depression of the scrape at x1/1 scale, and the nest elevation at x4/1 scale.

The 3D scans were processed using the open‐source program MeshLab v.2022.02 (Visual Computing Lab – ISTI – CNR, http://meshlab.sourceforge.net/) to extract only the height data and export the scans as .ply files (Simonov & Matantseva, 2020). These files were then imported into ImageJ using a custom script to create images containing each nest's *X*, *Y*, and *Z* (height) coordinates, with 1 pixel representing 1 mm (Schneider et al., 2012). Self‐occlusion from vegetation could result in missing *Z* values (0.07% of pixel values) these were replaced by using the surrounding median. Finally, ImageJ was used to label the different parts of each scan with ROIs (Regions of Interest), including the clutch (area of eggs), the nest (2.5x clutch surround), and the background (remaining area, not including clutch or eggs) see Figures 1 and 2. Instructions can be found within our Data S1 and on GitHub. For the null background scans, the average nest size was used to create a circular selection and the surrounding area was used for the background.

### 2.5 Nest 3D measures

Cross‐section maps of each nest were constructed by creating a circle selection (radius = 300 mm) centered on the clutch ROI. At each integer distance (radius = 0–300 mm), the mean *Z*‐value was measured and translated either to the minimum of the clutch (nest‐normalized) or the surrounding background (radius‐normalized) (see Data S1). These cross‐sections allowed for comparisons of the scans' peak nest, peak clutch, and trough heights and calculations of the nest's slope.

To quantify how rough or smooth the terrain at nest locations was at different spatial scales, we used methods similar to those used for 2D pattern analysis. We measured the “energy” (Standard Deviation, StdDev) of *Z* values at different spatial scales relative to the spatial frequency of the clutches in the following octaves (1/8x, 1/4x, 1/2x, 1/1x, 2/1x, 4/1x; Lindeberg, 2015; Michalis et al., 2017). *Z* energy represents the 3D topographic variation at the given spatial scale, with smaller spatial scales, for example, 1/8x, representing coarse surfaces such as grassy vegetation while larger spatial scales, for example, 4/1x, resulting from mounds or large tussocks. On average, clutches had a spatial frequency of 86 mm. The spatial frequency was calculated by using the square root of the clutch area. Energy maps for each spatial scale were made using the difference of Gaussians (DoG); subtracting each octave by 1.6x the same scale (Figure 2).

### 2.6 Clutch occlusion and visibility

**FIGURE 3:**
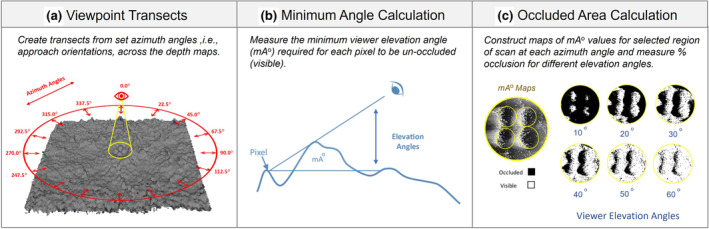
Phases for calculating clutch occlusion/visibility. (a) Create a cone of transects from each pixel in the clutch to observer locations in a series of azimuth angles. (b) Measure the minimum angle (mA°) required for each clutch coordinate/pixel to be un‐occluded by any other pixels along the transect, that is, visible. (c) Output the mA° as a map and threshold the map repeatedly using a matrix of viewer elevation angles to measure the percentage of the eggs visible or occluded for each angle. Lighter regions of the mA° map indicate sections that require a higher viewing angle to be visible.

For each depth map, occlusion maps were created for 16 different observer orientations around the azimuth of the nest, from 0° to 337.5°, in 22.5° intervals. To create these occlusion maps, we measured the shallowest or minimum elevation angle (mA°) required for each pixel in the clutch to be un‐occluded using a custom ImageJ script (see Figure 3). This was repeated for each of the 16 observer orientations. The resulting mA° maps could then be used to measure the percentage of the clutch occluded at specified elevations and azimuth angles. Elevation angles higher than a pixel's mA° indicate that the pixel is unoccluded or, in other words, visible. For observer elevation angles between 0.5°and 60°, in 0.5° intervals, we measured the percentage of the clutch visible. Percentage visibility at a given elevation angle was given as the mean percentage of pixels unoccluded across all 16 azimuth bearings, though the minimum, maximum, and deviation of visibility could also be measured. The horizontal distance required to achieve the viewing angles was calculated from different fixed observer heights: that of the average European red fox (0.4 m) and a matrix of increasing corvid flight heights (1.6, 3.2, 6.4, 12.8, 25.6 m). Flight heights were chosen as octaves relative to fox and human height (1.6 m) and based on the flower flight altitudes of foraging birds, observed even in higher flying raptors (Pfeiffer & Meyburg, 2022).

### 2.7 Camouflage metrics

Luminance Δ*S* and color Δ*S* (JND) of the clutch from its nest and background were modeled for corvid and fox vision under natural illumination as a metric of camouflage from background‐match with the mica toolbox (Jacobs et al., 1993; Martin, 2017; Moher Alsady et al., 2016; Vorobyev & Osorio, 1998). A greater Δ*S* value for luminance or color corresponds with a poorer match, with a JND of less 1–3 considered to be indiscriminable under normal viewing conditions. The Siddiqi method was used for Δ*S* luminance (Weber fraction 0.2) and the RNL model for Δ*S* color (Weber fraction of the most abundant cone of 0.05) (Lind et al., 2013; Moher Alsady et al., 2016; Pretterer et al., 2004; Siddiqi et al., 2004; Vorobyev & Osorio, 1998). For each observer, we used the most phylogenetically relevant known visual system as a model. These were the common peafowl Pavo cristatus (peak spectral sensitivities of 432, 477, 537, and 605 nm accounting for oil droplet and visual media absorption), for the corvid vision, and the red fox *Vulpes vulpes* (peak spectral sensitivities of 438 and 555 nm), for the fox vision (Jacobs et al., 1993; Malkemper, 2014; Malkemper & Peichl, 2018; Ödeen & Håstad, 2013). The common peafowl was chosen for corvid vision as it shares the VS (432 nm) sensitivity peak observed in corvids as opposed to the bluetit model commonly used for UVS systems.

Luminance Δ*S* and color Δ*S* values were measured for images acuity corrected, with the acuity view tool, for a given series of viewer elevation angles (1.875°, 2.5°, 3.75°, 5°, 7.5°, 10°, 15°, 20°, 30°, and 40°), when at the height of the model observers (Caves & Johnsen, 2018; van den Berg et al., 2020). As the heights were fixed, the total (hypotenuse) observer distance from the clutch was calculated using the height and viewing angle. The viewer elevation angles for corvid vision were then adjusted post‐hoc to the matrix of values used for clutch occlusion (1.6, 3.2, 6.4, 12.8, and 25.6 m) by calculating the elevation angle and horizontal distance required to produce the same total observer distance. For a given observer, not only does spectral sensitivity vary, but so too does the minimum angle with which they can resolve contrast in luminance or color information. Acuity correction was carried out using the known peak resolving power measured in cycles per degree (cpd), magpie *Pica* 33.33 cpd, and red fox *Vulpes vulpes* 8 cpd (Malkemper, 2014; Martin, 2017). Background luminance and spatio‐chromatic variation were also measured using the StdDev of the luminance and the sum StdDev of the RNL channels, respectively.

### 2.8 Statistical analyses

Statistical analyses were performed using R, version 3.6.3 (R Core Team, 2021). The *Z* energy metrics were treated as continuous variables and were log‐transformed so that residuals fit a normal distribution. To compare the effects of local site selection on background *Z* energy, we used linear mixed models with the lme4 package (Bates et al., 2014). The log (*Z*‐Energy) was given as the response variable, with the polynomial of spatial scale and the ROI (nest‐background or null‐background) as the fixed effects. To control for site and local effects, both the nest site and the nest ID for the nest and associated null were used as random effects following the formula:

$$\text{lmer}\;\left(\log\;\left(Z\;\text{Energy}\right)\sim \text{poly}{\left(\text{Scale},2\right)}^{*}\;\mathrm{ROI}+\left(1|\text{Site}\right)+\left(1|\text{NestID}\right),\ldots \right)$$

where ROI specifies nest vs null measurement, and NestID is the shared ID for the nest and its corresponding null. The effect of *Z* energy on the mean and standard deviation of nest temperature as well as the change in visibility (un‐occluded) with viewing angle was also tested using linear mixed models, with *Z* energy as the fixed effect. Time of year, county, and site were used as additional random effects for temperature analyses.

To compare the effects of management (crop, fallow, wet grassland, quarry and sheep‐grazed) on surrounding 3D variation as well as on luminance match and color match to the background, Tukey post hoc tests for pairwise comparisons were used. The metrics for *Z* energy, color match, and luminance match for the different visual systems were used as the response variables, while management was given as the fixed effect with site as a random factor. The effects of different metrics for camouflage on predation were analyzed using a binomial generalized linear mixed models with binary predation (no/yes) as the response variable and the metrics of camouflage (luminance Δ*S*, color Δ*S*, Background Luminance Dev, Background 3D Dev and Occlusion) as the fixed effect. For the random effects, the presence or absence of predation recorded at the site (PredatorPresent, no/yes) and the site ID were used:

$$\begin{array}{c} \text{glmer}\;(\text{Predated}\sim \text{CamouflageMetric}+(1|\text{Site})\\ +(1|\text{PredatorPresent}),\text{family}=\text{binomial}\ldots ). \end{array}$$

## 3 RESULTS

### 3.1 Nest site selection

**FIGURE 4:**
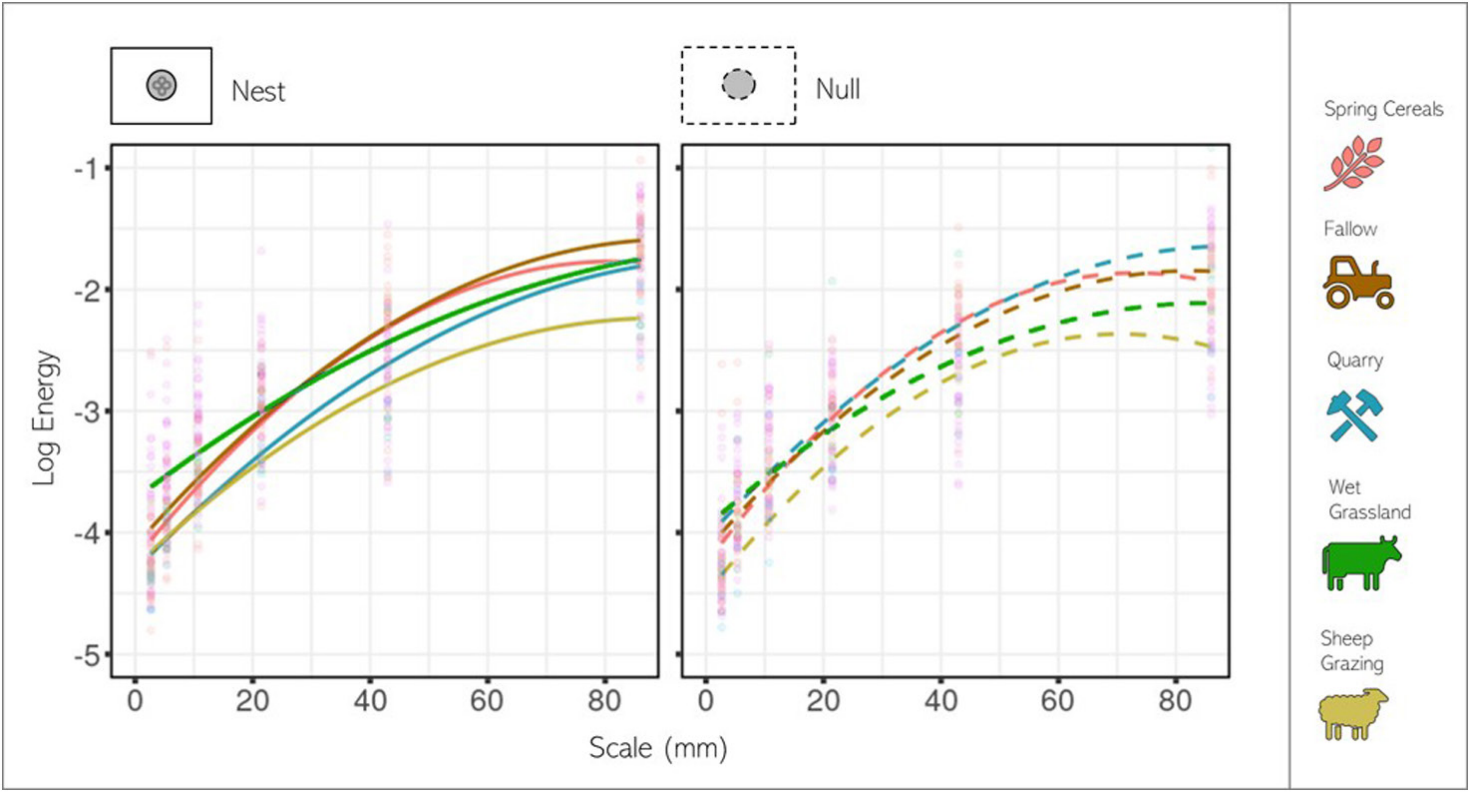
Comparison of log 3D (*Z*) energy at different spatial scales for the nest scans and null scans in different habitats, spring cereal, fallow, quarry, wet‐grassland and sheep‐grazing. The measured region of the scan is shown in white; the grey areas were excluded.

The *Z* energy of both null and nest‐site backgrounds increased with spatial scale, following a quadratic (scale^2^, β = −13.96, SE = 0.16, *p* < .001 | scale, β = 24.12, SE = 1.15, *p* < .001); see Figure 4. As would be expected if lapwing were selected for microhabitats with increased 3D complexity, lapwing nest surrounds possessed greater 3D variation across all spatial scales (nest, β = 2.90, SE = 0.12, *p* = .004) compared to their null, and variation increased with spatial scale at a faster rate for nest sites at the smaller spatial scales (nest:scale^2^, β = 2.621, SE = 0.04, *p* = .009 | nest: scale, β = −2.029, SE = 0.13, *p* = .042). Variation in *Z* energy was not found to effect the nest mean or standard deviation of clutch temperature, irrespective of spatial scale. Only time of year significantly effected nest temperature, with nests later in the breeding season being warmer (TimeOfYear, β = 2.621, SE = 0.02, *p* < .001).

### 3.2 Management effects

Post hoc comparison of site management strategies showed the nest sites of sheep grazed fields had significantly lower 3D variation compared to other sites, while wet grassland sites had significantly greater 3D variation (see Data S1). For spatial scales smaller than the size of the clutches, *Z* energy originated from deviations in height between small vegetation (grasses) or from the substrate (large stones, gravel). The *Z* energy of pastoral nest sites at larger spatial scales was more similar to that of the arable sites than their null sites, except for at sheep grazed sites. At scales greater than the size of the nest, high energy resulted from large clumps or mounds of weedy vegetation, trampling, and sloping terrain (hills). On average, clutches were elevated 4.5 cm (±2.4 cm) above their local surroundings. There was no significant difference in nest elevation between management types. Nest elevation was instead predicted by the *Z* energy of the surroundings (*Z* energy: elevation, β = 2.894, SE = 53.816, *p* = .005).

### 3.3 Viewing angle, clutch occlusion and camouflage

**FIGURE 5:**
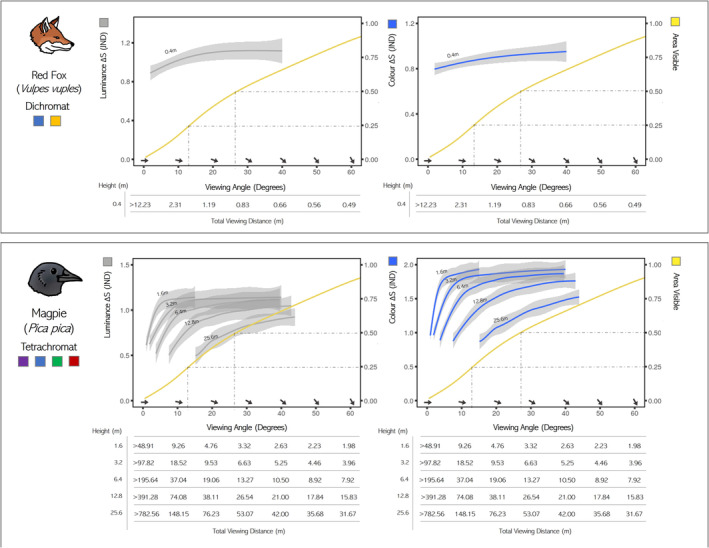
The effect of viewing angle and distance on both the percentage of the clutch un‐occluded by surrounding structures (yellow curve, right hand axis) and the luminance Δ*S* (grey)/color Δ*S* (blue) (left hand axes) from the fixed predator heights of a red fox (height = 0.4 m) and a magpie at different octaves of heights [height = (1.6, 3.2, 6.4, 12.8, 25.6) m]. The X axis shows both the viewing angle and the total distance (below) required to be at that viewing angle for the different fixed heights. The dashed lines show the angle required for 25% and 50% of the clutch area to not be occluded.

The percentage visibility (un‐occluded) of the clutch (eggs only) increased with the observer's viewing angle in a sigmoid fashion. On average, a viewing angle of 15° elevation [equivalent horizontal distance: Fox 1.5 m, corvid (6.0, 11.9, 23.9, 47.8, 95.5 m)] was required for 25% of the clutch to be un‐occluded and an angle of 27° [horizontal distance: Fox 0.8 m, corvid (3.14, 6.2, 12.6, 25.1, 50.2 m)] for 50% (Figure 5). Increased 3D energy across spatial scales increased nest occlusion at low viewing angles (0 °–40 °). Spatial scales below the clutch size (scale of coarse vegetation) had the greatest effect on occlusion compared to larger scales (sum smaller scales, β = 130.40, *p* < .001 | sum larger scales, β = 99.37, *p* < .001); see Data S1 for figures.

The JND color and luminance difference of the clutches from the local surround were in line with those of highly camouflaged animals at less than 2 JND (Fox Vision, Lum Δ*S* mean 1.10 ± 0.02 SE | Col Δ*S* mean 0.85 ± 0.02 SE) (Corvid Vision, Lum Δ*S*, mean 0.9 ± 0.02 SE | Col Δ*S* mean 1.58 ± 0.02 SE). Clutches were of a better color match to bare crop and fallow sites as opposed to the vegetated wet grassland sites for both visual systems (Sussex vs Hampshire: Corvid Vision Col Δ*S* β = −6.33, SE = 0.87, *p* < .0001) (Sussex vs Hampshire: Fox Vision Col Δ*S*, β = −7.43, SE = 0.80, *p* < .0001). Color Δ*S* and luminance Δ*S* followed a negative exponential with increasing viewing angle (Figure 5). As the viewing distance increased, the viewing angle and percentage of the nest visible decreased as the observer's height was fixed. The decrease in Δ*S* from distance caused by visual acuity was ubiquitous after the majority of the clutches were occluded from most predator heights. For an increase in horizontal distance to drop color Δ*S* and/or luminance Δ*S* by just 0.1 JND, the clutches would already be 75% occluded. The exceptions were for corvid vision from a height of 12.8 m (22.5 ° for −0.1 JND) and 25.6 m (32.5 ° for −0.1 JND).

### 3.4 Nest predation

**FIGURE 6:**
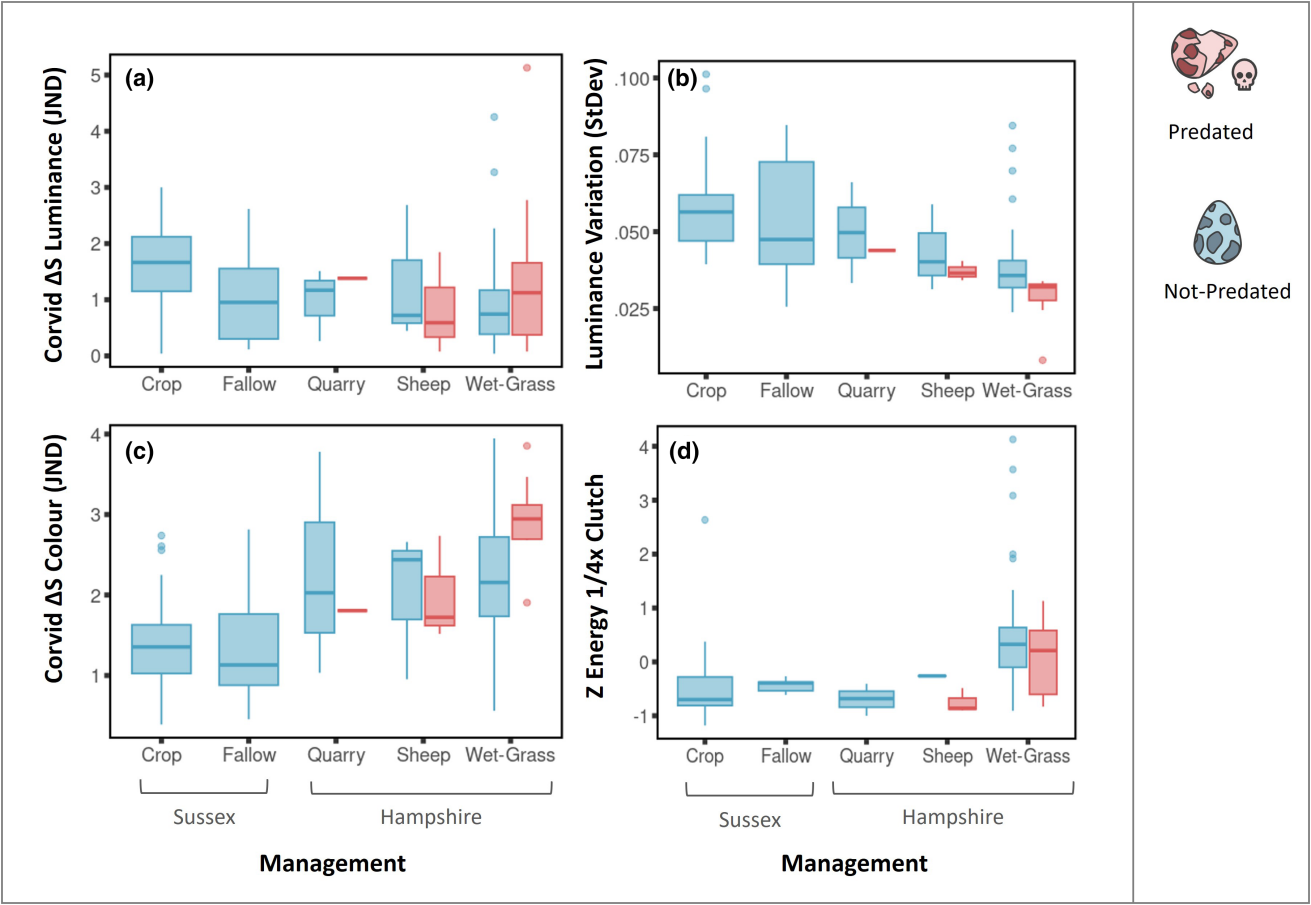
Interactions between camouflage metrics and nest outcomes for each management type. The camouflage metrics are as follows: (a) the luminance difference of the clutch from the background in corvid vision; (b) the color difference of the clutch from the background in corvid vision; (c) the luminance variation of the clutch's surrounding background; and (d) the clutch's background *Z* energy, that is, the topographic variation. Outcomes are predated or not‐predated.

Over the 2 years we sampled the Avon Valley and Sussex Sites we photographed and scanned 115 lapwing nests, however 29 scans were lost due to equipment damage in 2022. Of the nests photographed, 13 were predated (8 in 2021, 5 in 2022). The proportion of nests predated varied widely between county and site, with no predation events of photographed or scanned nests recorded in the Sussex sites. Though nest predation of unscanned or photographed nests did occur (8 total in Sussex). Predation was the most common cause of nest failure across all sites, followed by abandonment (7 abandoned, 4 others). On average, predated nests had poorer color match (predated, mean = 2.53, StdDev = 0.72) (un‐predated, mean = 1.83, StdDev = 0.85) and lower surrounding luminance variation (predated, mean = 0.29, StdDev = 0.56) (un‐predated, mean = 1.12, StdDev = 1.05), see Figure 6. However, none of the camouflage metrics (visibility, luminance match, color match, background luminance complexity, or background color complexity) were able to significantly predict nest failure from predation. The results for occlusion were unchanged when using the azimuth angle with the minimum and maximum visibility. Likewise, comparisons only including Hampshire failed to find any prediction of predation from camouflage.

## 4 DISCUSSION

Here, we provide one of the first empirical measures of animal occlusion from different predator viewing angles and the first use of observer height as a factor for acuity modeling. Measurements of camouflage from arrays of different distances with acuity views and related modeling tools are increasingly being used in publications on the functions of animal color patterns (Barnett et al., 2018; Nokelainen et al., 2021). However, studies frequently fail to account for occlusion in determining whether or not the viewing distances used for visual models are biologically relevant. Our results show that “openness” at a human scale does not reflect openness at scales relative to the nests (Allen et al., 2011), with nest occlusion being more likely to limit detection distance than visual acuity. Especially when viewed at the height of terrestrial predators, where the scales of the clutches and observers render the 3D scene more akin to a closed habitat, the bowl shape of the nest occluding the clutches at low angles. The ability to obtain a broader range of viewing angles for objects unobstructed by local structures and independent of an animal's physical height or habitat topography is a likely driver of the increased acuity observed in aerial predators. Short terrestrial predators, on the other hand, should not be selected for visual acuities capable of segmenting objects further than they are capable of observing unobstructed. Previous work investigating the search behavior of foxes and domestic dogs trained to find nests has found them to have a short localization distance of <2 m (Seymour et al., 2003; Storaas et al., 1999). Both our Δ*S* measurements and occlusion measures support this observation. Discrimination of the clutch outline at short distances is likely to be the mechanism of egg detection for most clutches, barring the few with unusually poor background matches. Clutches with greater visibility (less vegetated) were also found to have a better color match in the corvid visual model. Whether these differences in match were due to greater selection intensity when less occluded or limitations in the avian egg color palette's ability to match live vegetation is difficult to disentangle with our current dataset (Hanley et al., 2015).

Previous research on landscape effects on lapwing nest success has shown that increased proximity to taller ground vegetation, being at a greater distance from the tree line, and having surrounding bodies of water decrease the risk of nest predation (Kaasiku et al., 2022; Laidlaw et al., 2017). The lapwings within our study system were found to nest preferentially in local habitats with greater 3D variation at scales greater than the size of the clutch. Habitats that feature depressions and topography (plough, cattle, and horse grazing) with similar scales to their nests should decrease lapwing predation by increasing the amount of visual noise and clutter at scales similar to nests (Swaisgood et al., 2018). Existing guidelines for creating suitable lapwing nesting sites, promoted by UK conservation organizations (e.g., RSPB, BTO, and GWCT), recommend fields with short patchy vegetation in pastoral sites (Ausden & Hirons, 2002; Smart et al., 2013). Analysis of lapwing habitat structure with our 3D scans supports this preference for patchy local sites with 3D variation greater than the scale of their nests. These results also emphasize previous work advising the avoidance of grazing species that create homogenous and flat vegetation, such as sheep (Winter et al., 2005).

The null scans for the arable sites were found to be more similar to than those of the nests than the pastoral sites. Ploughed sites also offered better color match, luminance complexity, and local 3D variation match to the lapwings' nests. While not significant, these sites had the lowest proportion of predation but were also under intense predator control. Northern lapwing populations have long been associated with spring cropland throughout Eurasia (Galbraith, 1988; Salek & Cepáková, 2006). Selection of these habitats has been thought to be and is likely driven by the large‐scale match to the locally preferred background 3D and color features found naturally within wet grassland. Nesting preference at these sites may be suboptimal for survival at later stages of their life history, acting as a sensory or ecological trap with greater chick predation and lower food availability present within these sites (Baines, 1990; Schekkerman et al., 2009). Furthermore, the potential advantages or disadvantages of broadscale selection of arable or local selection of 3D complexity and color match could be masked by predator control rather than indicative of their adaptive value.

Modeling occlusion with handheld 3D scanners can be a useful tool for estimating an object's visibility; however, it does not account for taller features at greater distances. The nests of the sampled lapwing were found in fields without much obstruction except at the boundaries (hedgerows & forests) (MacDonald & Bolton, 2008). Other UK ground‐nesting waders, for example, Eurasian curlew *Numenius arquata* and redshank *Tringa totanus*, and populations of lapwing in more forested areas are more likely to have visibility influenced by structures further from the nest than in our 3D scans. Using large‐scale LIDAR scans in conjunction with fine‐scale scans could provide a broader map of the visibility and cover of nests (Lone et al., 2014). 3D scanning and color‐calibrated photography could also be useful for assessing other aspects of nest microhabitat selection, such as the effects of exposure to the sun throughout the day and nest albedo on the average nest temperature and nest temperature fluctuations (Lindberg & Grimmond, 2011). Over a larger geographic or climate range or within warmer climates, more occluded or geometrically complex nests could be more important for thermoregulation than just crypsis (Amat & Masero, 2004).

Observation from lower visual angles is likely to influence background match and edge disruption, yet little is known about how its interactions with camouflage should be measured. Partial and self‐occlusion will reduce the visible area of the clutch and mask recognizable features such as the clutch's shadow and edge (Lovell et al., 2013; Webster, 2015). Meanwhile, changes in background scene statistics from the change in orientation of shapes, particularly vegetation, and spatio‐chromatic complexity across the horizon may also affect the detection of ground‐nesting bird eggs and other camouflaged objects. Future work should consider measuring camouflage in the presence of obstructions and/or from different visual angles. In particular, experiments measuring the survival of sedentary objects, such as eggs or model animal targets, where object motion and changes in the local 3D environment are less prevalent. The use of 3D multispectral models or color‐calibrated video cameras may also provide potential alternate technological solutions to the challenges of measuring visibility from multiple viewing angles (Miller et al., 2022; Vasas et al., 2022). However, these methods are slower and more computationally expensive than our 3D phone scans. Finally, our study serves as a reminder of how occlusion is integral to understanding the distances at which visual systems can interact with natural objects and the adaptations required to break camouflage from biologically relevant distances.

## AUTHOR CONTRIBUTIONS

**George R. A. Hancock:** Conceptualization (equal); formal analysis (lead); investigation (lead); methodology (equal); visualization (lead); writing – original draft (lead). **Lizzie Grayshon:** Investigation (supporting); methodology (supporting). **Ryan Burrell:** Investigation (supporting); methodology (supporting); writing – review and editing (supporting). **Andrew Hoodless:** Conceptualization (equal); funding acquisition (equal); methodology (equal); supervision (equal); writing – review and editing (equal). **Innes Cuthill:** Conceptualization (equal); funding acquisition (equal); methodology (equal); supervision (equal); writing – review and editing (equal). **Jolyon Troscianko:** Conceptualization (lead); funding acquisition (lead); methodology (equal); supervision (lead); writing – review and editing (equal).

## FUNDING INFORMATION

NERC GW4+ NE/S007504/1 funded George R. A. Hancock in a CASE partnership with the Game and Wildlife Conservation Trust. Jolyon Troscianko was funded by a NERC Independent Research Fellowship NE/P018084/1.

## CONFLICT OF INTEREST STATEMENT

The authors declare no conflict of interest.

## Supporting information


Data S1:

## Data Availability

The dryad doi: https://datadryad.org/stash/share/2yJJ6xuGiKF7Ey6H2y1rAwyqJVGYFdBgFisTb4NN34I. All data and plots for the main text and supplementary material can be found within our dryad archive. ImageJ scripts for running RNL and 3D analyses with the MICA toolbox and ImageJ can be downloaded from our GitHub: https://github.com/GeorgeHancock471/3D_RNL_Tools.
