# Supplementary material for: Habitat geometry rather than visual acuity limits the visibility of a ground‐nesting bird's clutch to terrestrial predators
Source: Ecol Evol. 2023 Sep 14;13(9):e10471. doi: 10.1002/ece3.10471 (PMC10501817; doi:10.1002/ece3.10471)
Supplement: Supplementary file 1 — Data S1: [file ECE3-13-e10471-s001.docx]

# **Supplementary Information for “****Habitat geometry rather than visual acuity limits the visibility of a ground-nesting bird's clutch to terrestrial predators”.**

**Authors:** George R.A. Hancock, Lizzie Grayshon, Ryan Burrell, Innes Cuthill, Andrew Hoodless, Jolyon Troscianko

# SI 1: Creating and measuring depth maps.

## Installation:

Download the 3D_Depth_Measures plugins from [our GitHub](https://github.com/GeorgeHancock471/3D_RNL_Tools) (Schneider et al., 2012)

Paste the plugins into the /plugins/ directory of ImageJ.

## Scanning and .ply Generation:

*Matterport Scans*


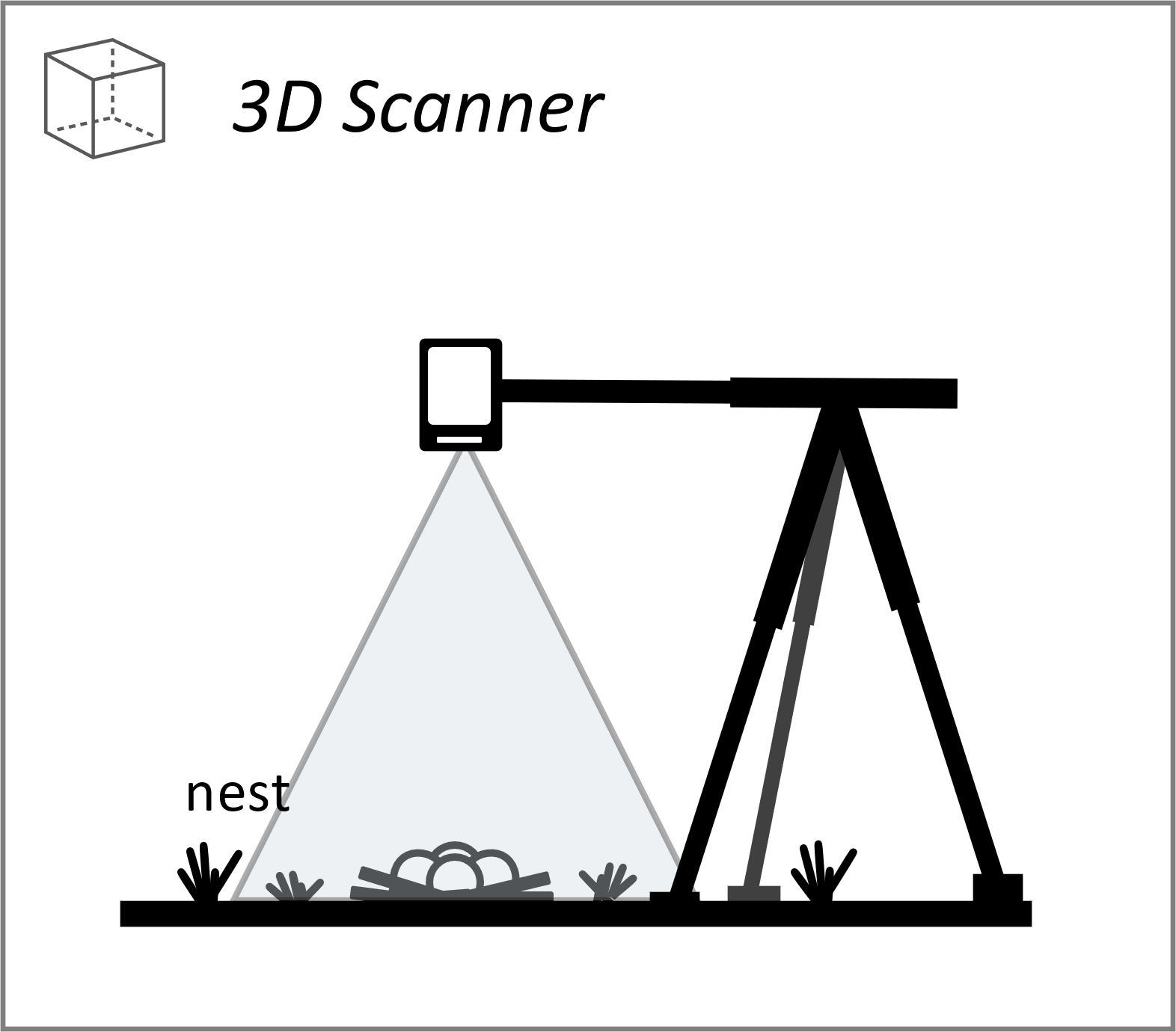
**1. Scans**

All 3D scans should be taken from a flat polar (vertical) viewing angle of 0^o^ and from the same viewing height. 3D scans are more easily taken under diffuse lighting conditions where the generated point cloud image is more visible.

**2. Extract .ply Files**

Copy .ply files from smartphone and paste the files into separate folders, each with an identifiable label. E.g., scan_23.03.21_06/

**3. Normalise .ply Files**

Open each .ply using MeshLab v.2022.02. Then export the .ply with no additional information (just the point cloud).

- File/Export Mesh As…

*Photogrammetry Scans*


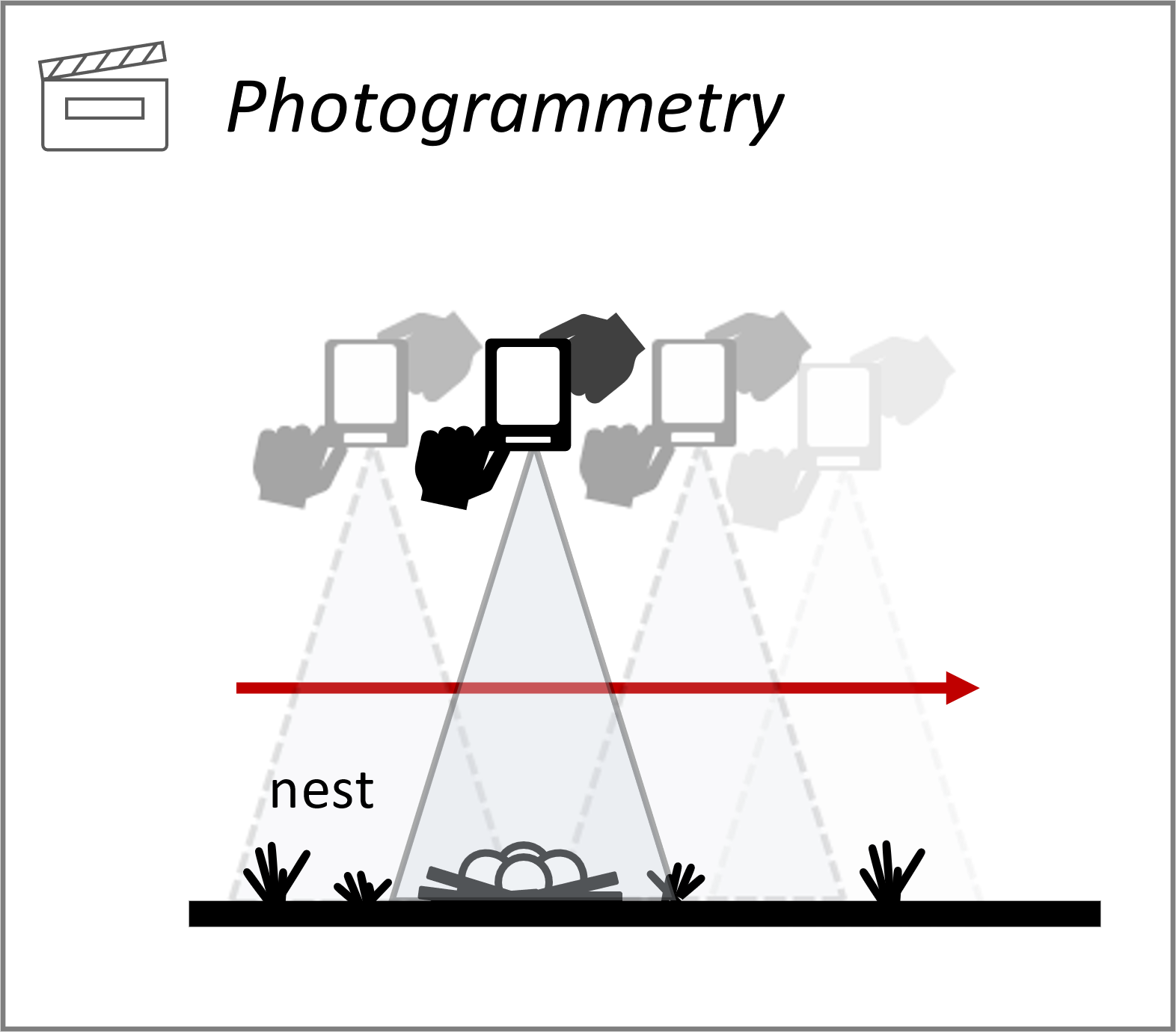
**1. Videos**

Take a video of the area with the camera held perpendicular to the direction of motion to maximise the area recorded. Each video should contain a scalebar of known dimensions. You will not be able to read the increments from the scan.

**2. Photogrammetry**

Videos can be converted to a stack of images for photogrammetry with keyframe selection or the VLC frame plugin. Images can then be converted to .obj 3D scans with the open-source software Meshroom-2021.1.0 (<https://alicevision.org/#meshroom>). Note a NVIDIA CUDA-enabled GPU is recommended for using Meshroom.

**3. Scaling**

Open the each .obj scan using MeshLab v.2022.02. Make sure that the x, y and z axis are correct. The swap axis use ‘*filter / Normals, Curvatures and Orientation / Transform: Flip and/or swap axis’*

Make sure that the scale matches that of the scale bar. Draw the measuring tool as a line over the scale bar. This will give you the scale meshlab thinks the object is in metres. Calculate the scale factor by dividing the actual scale by the meshlab scale. Then use *filter / Normals, Curvatures and Orientation / Transform: Scale’* and adjust the scale with the scale factor.

**4. Export as normalized .ply**

Export the .obj as a .ply file with no additional information (just the point cloud).

- Select Saving Options: [•] None & Binary encoding [✓]

You should export the normalised image with a new name e.g., point_cloud_normalised.ply into an identifiable folder E.g., scan_23.03.21_06/

## Depth Maps:

Open ImageJ with the DepthMeasures plugins installed.

Run plugins /0 Create DepthMaps / Batch Create Maps.

This plugin allows you to loop open normalised point clouds and convert them to .tif 2D images where x=x, y=y and the pixel value (v) = z.

You can then manually label regions of interest (ROIs) or use a custom script for labelling. Example ROI scripts are provided for backgrounds with (ROIs_Clutch) and without nests (ROIs_Null). Which were used for *“Measuring nest occlusion with 3D scanners and its implications for lapwing nest camouflage”.*

You should in the end have a folder of folders containing a normalised.ply file and a depth_map.tif .

## Measures:

The measurement scripts can be applied to any folder with sub folders of ‘depth_maps’.

Measures are output as a /t delimited text files within the selected folder.

*ROI Measures (1 Depth ROI)*

You can either measure the mean,min,max and dev for specified ROIs or measure the Difference of Gaussian Energy at specified spatial scales. We recommend using scales relative to the avg size of an ROI.

*Transect Measures (2 Depth Transect)*

Measures the min, max and avg z value for integer radius from 0 – R, where R is the specified maximum radius.

The centre point of the circle is that of a selected ROI.

*Visibility Measures (3 Depth Visibility)*

Measuring clutch visibility requires two steps to be completed. NB: this method was designed for circular objects such as eggs.

Step 1. Generate visibility maps for a specified selection, azimuth angle interval and radii.

*You will need to select the output area for the ROI (saved selection) and the radius from which occluding pixels are measured.*

*You can also specify an ROI from which occlusion is ignored e.g., to eliminate self-occlusion, useful for comparing occlusion from surround vs occlusion from self.*

*Visibility maps are output as .tif files labelled for the chosen ROIs and azimuth interval.*

Step 2. Measure visible area of selected visibility maps.

*You will need to specify the same ROI as the saved selection.*

# SI 2: Nest transects.


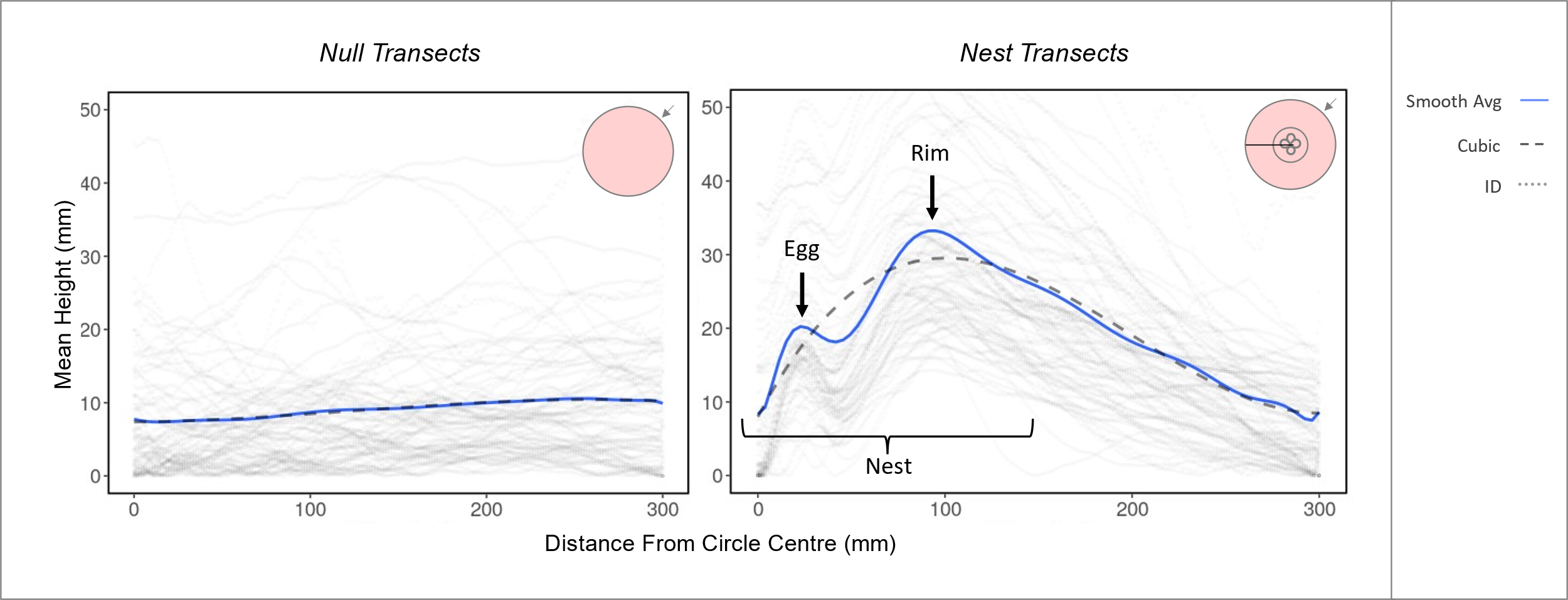


SI Figure 1. Transects of 3D scans. Left shows the transects of the nest-less scans, right shows the transects for the nest scans. For each transect the smoothed average (blue), cubic (grey dashed) and the individual trend lines (grey dotted) are shown. The peaks for the eggs and rim of the nest are marked by arrows.

Transects of scans showed a consistent pattern of variation for the nest sites. With two peaks, the first peak being from the eggs and the second from the rim of the scrape (See SI Figure 1). To test the significance of the two peaks the height and distance metrics were normalised and separated. First the data frame for the null and nest scans were reduced to only include distances at 50mm intervals. Linear mixed models were used with the nest ID as a random factor. *lmer (Normalised-Height~ poly(Normalised-Distance,2) + 1|NestID),…)* (Bates et al., 2014; R Core Team, 2021)

Nest scans showed a significant cubic relationship with distance (distance^3^, β= 7.675, SE = 1.1272, p<0.0001 | distance^2^, β= -5.7808, SE = 1.144, p<0.0001 | distance, β= 9.743, SE = 9.4520, p<0.0001) with height increasing then decreasing with distance from the centre of the nest. No significant trend was observed for the null scans. For the egg peak, the nest dataset was reduced to distances of <= 50mm from the centre point and at intervals of 5mm. The egg peak was significant with a quadratic relationship with distance (distance^2^, β = -24.68, SE = -0.30072, p<0.0001 | distance, β = 26.95, SE = 0.98965, p<0.0001).

# SI 2: Occlusion modelling

Nest occlusion was modelled at 16 different orientations at 22.5^o^ intervals using the measure visible area plugins (see GitHub). The visible area was then calculated at 0.5^o^ intervals from 0.5 – 70 degrees.


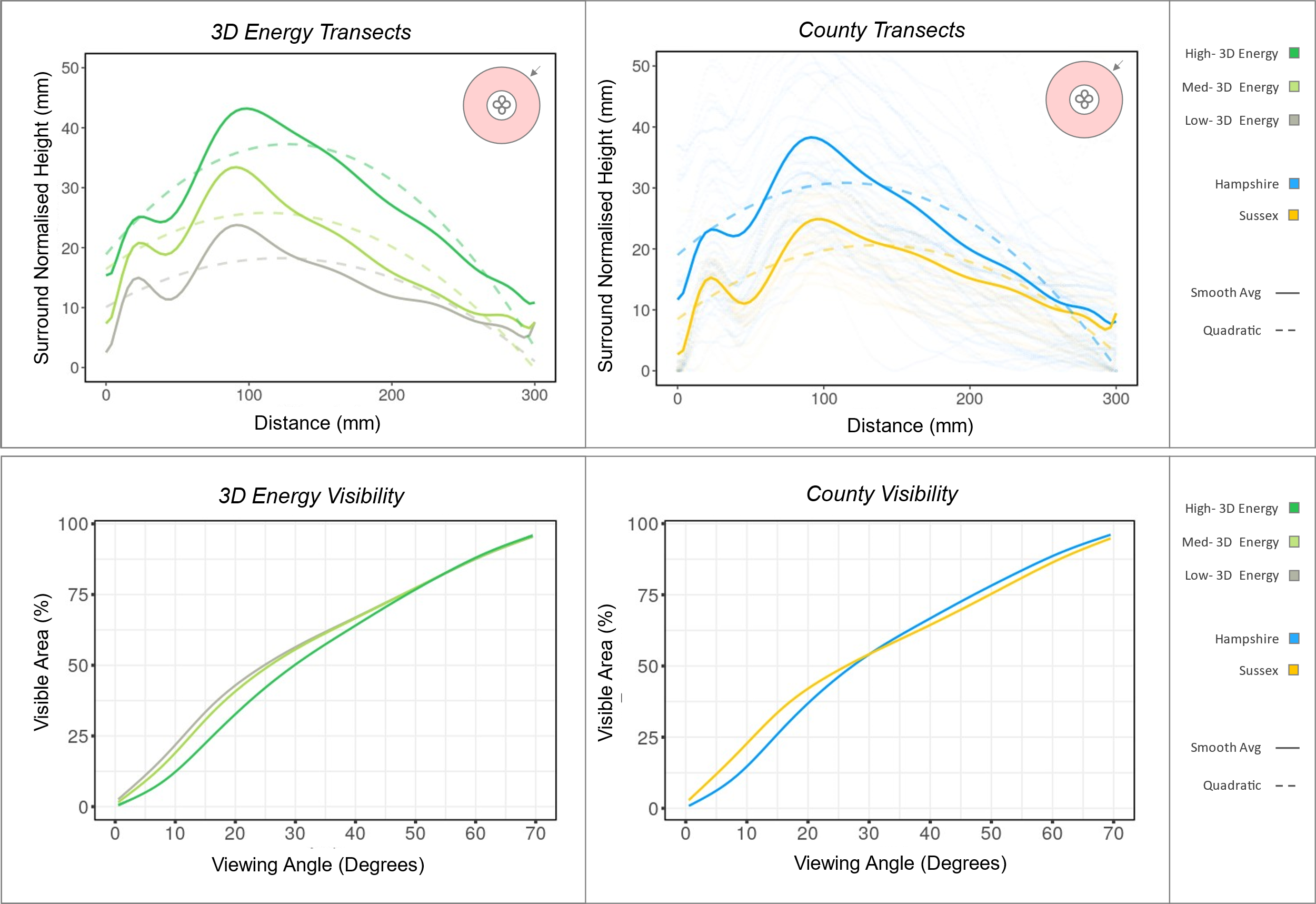


SI Figure 2. Above shows transects of the 3D scans normalised to the minimum of the nest surrounds, below shows the percentage visible area at given distances. Left shows the data split by difference of Gaussian energy at scales less than or equal to the clutch size, while the right shows the data split by county. 3D energy was categorized by splitting into 3 separate quantiles.

We compared occlusion between nests surrounds with higher 3D energy at scales less than or equal to the clutch size, as well as between the two counties (SI Figure 2). Nests with higher 3D energy at these scales were more locally elevated (3D Energy: β = 216.3, SE=24.95, p<0.001) and were less visible (3D Energy: β = -0.8673, SE=0.02031, p<0.001). While nests in Sussex were also on average less locally elevated (Sussex: β = -7.54, SE=2.21, p<0.001) and occluded (Sussex: β = 0.07029, SE= 0.02337, p= 0.00714), but only at angles less than 30 degrees. When factoring local nest elevation, the visibility of the nests further decreased with horizontal viewing distance and background 3D energy but maintained the same relationship with viewing distance (SI Figure 3).


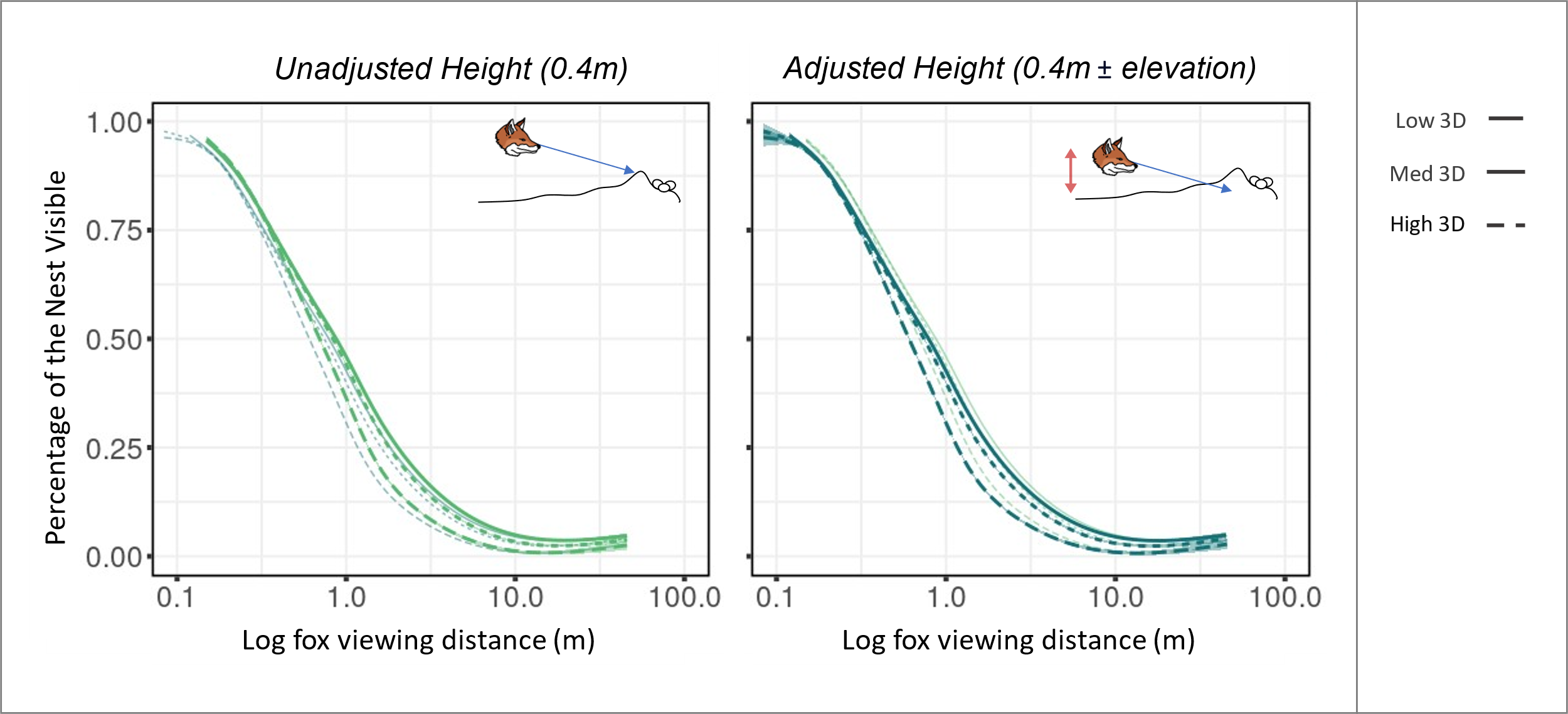


SI Figure 3. Transects of nest visibility at different viewing distances from a fox’s height. Left shows unadjusted viewing height and right shows the viewing height adjusted for the difference in elevation. Translucent lines show the position of the opposite plot. Line dashes group the nests by the quantiles of difference of Gaussian energy at scales less than or equal to the clutch size.

# SI 3: 3D energy comparisons

| Pairwise Comparison | *β* | SE | p. value |
| --- | --- | --- | --- |
| Crop-Fallow | -0.973 | 0.0800 | 0.8675 |
| Crop-Quarry | +1.597 | 0.1075 | 0.5001 |
| Crop-Sheep | +3.239 | 0.0862 | 0.0112 * |
| Crop-Wet Grassland | -3.950 | 0.0456 | 0.0009 *** |
| Fallow-Quarry | +2.005 | 0.1244 | 0.2651 |
| Fallow-Sheep | +3.351 | 0.1066 | 0.0078 *** |
| Fallow-Wet Grassland | -1.319 | 0.0775 | 0.6794 |
| Quarry-Sheep | +0.837 | 0.1285 | 0.9190 |
| Quarry-Wet Grassland | -3.329 | 0.1056 | 0.0083 *** |
| Sheep-Wet Grassland | -5.477 | 0.0839 | <.0001 *** |

SI Table 1. Tukey-Post Hoc test output for pairwise comparison of 3D energy of the nest surrounds between management schemes. Significant results are marked with * for p<0.05. Significant negative T values are marked in red and positive values are marked in blue.

# SI 4: Predation results

No predation events were observed in any of the photographed Sussex nests. The majority of predation events were recorded in wet grassland, while the sheep grazed management had the highest proportion of predation of any management type.


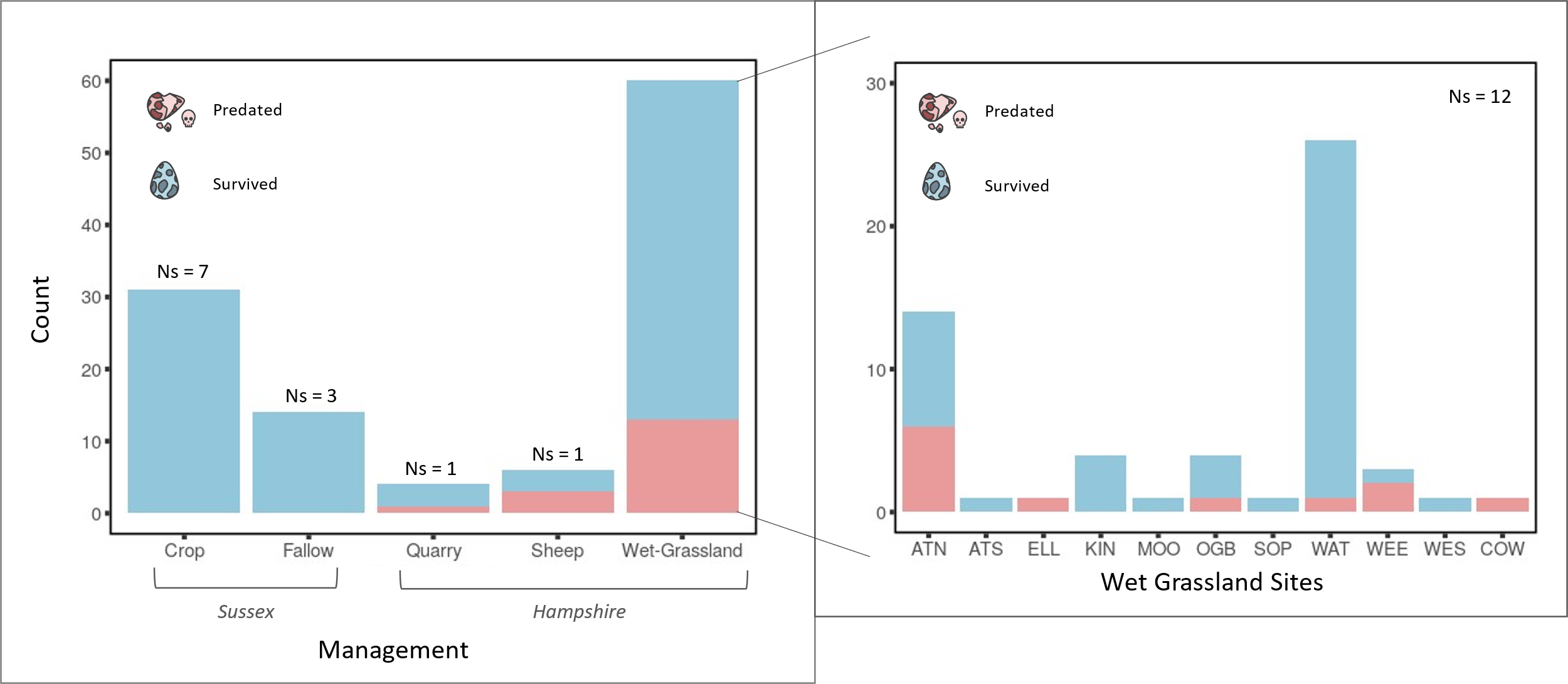


SI Figure 4. The number of nests and the proportion predated per management type. Ns denotes the number of sites of a given management type. The wet-grassland sites are separated as their individual site.

# Supplementary References

Bates, D., Mächler, M., Bolker, B., Walker, S., 2014. Fitting linear mixed-effects models using lme4. ArXiv Prepr. ArXiv14065823.

R Core Team, 2021. R: A language and environment for statistical computing. R Foundation for Statistical Computing, Vienna, Austria, 4.0. 5.

Schneider, C.A., Rasband, W.S., Eliceiri, K.W., 2012. NIH Image to ImageJ: 25 years of image analysis. Nat. Methods 9, 671–675.
